# Depression and anxiety among patients with Parkinson’s disease: A cross-sectional study in a Saudi population

**DOI:** 10.1097/MD.0000000000048538

**Published:** 2026-05-01

**Authors:** Sohaila Alshimemeri, Mohammed A. Aljaffer, Ayedh H. Alghamdi, Nouf F. Alshammari, Naif Alsaber, Rakan S. Alromayan, Ahmad H. Almadani

**Affiliations:** a Neurology Unit, Department of Medicine, College of Medicine, King Saud University, Riyadh, Saudi Arabia; b Department of Psychiatry, College of Medicine, King Saud University, Riyadh, Saudi Arabia; c SABIC Psychological Health Research and Applications Chair (SPHRAC), Department of Psychiatry, College of Medicine, King Saud University, Riyadh, Saudi Arabia; d Department of Psychiatry, Eradah Complex for Mental Health, Riyadh, Saudi Arabia; e Department of Neurology, King Abdulaziz Medical City, Ministry of National Guard and Health Affairs, Riyadh, Saudi Arabia; f College of Medicine, King Saud University, Riyadh, Saudi Arabia.

**Keywords:** anxiety, depression, GAD-7, Parkinson’s disease, PHQ-9

## Abstract

Depression and anxiety are among the most common comorbidities in individuals with Parkinson’s disease (PD). Yet their prevalence and contributing factors in Saudi Arabia are poorly examined. This study aimed to estimate the prevalence of depression and anxiety among patients with PD in Saudi Arabia, along with contributors, demographics, and clinical correlates. A cross-sectional study was conducted in Riyadh, Saudi Arabia, in which 130 patients diagnosed with PD completed the validated Arabic versions of the Patient Health Questionnaire-9 (PHQ-9) and Generalized Anxiety Disorder-7 (GAD-7) to ascertain the presence of depression and anxiety, respectively. Sociodemographic, patient-related factors, and clinical data were also collected and analyzed in conjunction with the aforementioned scales using univariate and multivariable logistic regression. Depression and anxiety were present in 67.7% and 62.3% of participants, respectively. Compared with the age group 41–50 years, the 51–60 and 61–70 groups were less likely to be depressed or anxious (*P *= .013 and *P *= .008). Lower education (high school or less) was associated with both depression and anxiety (*P *= .021 and *P *= .017). Anxiety (GAD-7) was associated with a history of major depressive disorder (*P *= .003). Longer PD duration was associated with higher odds of both anxiety and depression (*P *≤ .001). Our study shows a high prevalence of anxiety and depression in patients with PD, particularly among those with longer disease duration and lower educational attainment. Routine neuropsychological screening and early multidisciplinary care are warranted.

## 1. Introduction

Parkinson’s disease (PD) is a common neurodegenerative disease that predominantly presents with motor features such as bradykinesia, rigidity, tremors, and postural instability, with resultant functional deterioration and potential loss of independence.^[1]^ A 2020 review estimated that 9.4 million people worldwide are living with the condition, and the prevalence has reportedly doubled over the past 20 years.^[2,3]^ According to the 2019 Global Burden of Disease (GBD) project, the age-standardized point prevalence of PD in the Middle East and North Africa (MENA) region was 82.6 per 100,000 population.^[4]^ Prevalence rates in Saudi Arabia are less clear, with the majority of the literature citing the estimates from the 1993 Al-Thugbah study, which reported prevalence rates of 27 per 100,000; this crudely corresponds to 9720 cases of PD in Saudi Arabia.^[5]^ However, it is likely that this figure is an underestimation due to factors such as population growth and potentially improved diagnosis and awareness of PD since the time of the study. It is also important to note that in PD, motor features may overlap with items included in commonly used depression and anxiety rating scales, which can influence symptom measurement and prevalence estimates.^[6]^

Secondary psychiatric manifestations are notably common in PD. Among these are depression and anxiety, which are some of the most common mental health conditions that significantly impair daily functioning.^[7,8]^ The psychosomatic effects of anxiety and depression in PD are not merely secondary symptoms but are closely intertwined with the disease’s progression, as they can exacerbate motor symptoms and impede therapeutic efficacy.^[9]^ These psychosomatic effects highlight the need for regular screening and comprehensive management strategies for patients with PD.^[9,10]^ The prevalence of depression among those with PD is significantly higher than in age-matched controls,^[11]^ with an estimate of 38% compared to a lifetime depression risk of 15–18% in the general population.^[12]^ Among patients with PD, the prevalence of anxiety is approximately 25% versus 10% in the general elderly population. Comorbid depression occurs in around 14% of PD patients.^[13]^

Higher rates of depression and anxiety in PD patients compared to age-matched controls have also been reported in the Middle East across different regions with relatively comparable proportions. For instance, an Egyptian study of 64 participants reported that 31.3% of PD patients had associated depression, while 40.6% had experienced anxiety; comorbid depression and anxiety were found in 23.4% of the participants.^[8]^ Supporting these findings, a cross-sectional study conducted in Lebanon showed that 46% of enrolled PD patients were subsequently diagnosed with depression. These findings were particularly pronounced in female patients.^[14]^ Despite the aforementioned association with moderate PD, the risk of depression is greater as the disease advances. An Algerian study reported rates of 66.18% and 69.70% for depression and anxiety, respectively,^[15]^ whereas in Tunisia rates of 38.3% and 27.8% were reported.^[16]^ Depression rates of 47.9% and 44% were reported in Morocco and Iran, respectively.^[17,18]^

In addition, a recent study found the prevalence of depression and anxiety in those with PD in Saudi Arabia to be 84.4% and 73.9%, respectively. However, the small sample of 46 patients may have led to an overestimation of the prevalence.^[19]^ The heterogeneity of the prevalence estimates of psychiatric manifestations in PD probably reflects several factors, including differing diagnostic techniques, sociodemographic characteristics, and potential cultural factors.^[20,21]^

Despite the clear heightened prevalence of anxiety and depression among individuals with PD, these manifestations have not yet been adequately studied in a Saudi population. This knowledge gap, along with the harmful effects of these comorbidities, prompted the present study. A better understanding of the estimates of depression and anxiety, along with culture-specific contributors in the PD population, could guide clinicians in initiating regular screening practices and enhance understanding of these conditions as they relate specifically to the Saudi PD population. This study aims to estimate the prevalence of depression and anxiety in those with PD and examines the potential clinical and demographic factors that may play a role in their development.

## 2. Methodology

### 2.1. Study setting, design, and participants

This cross-sectional study was conducted at King Khalid University Hospital (KKUH) in Riyadh, Saudi Arabia. Participants were recruited from the KKUH Parkinson’s disease clinic and the Saudi Parkinson’s Society. Inclusion criteria were a clinical diagnosis of PD and age ≥ 40 years. Exclusion criteria were any psychiatric disorder other than depression or anxiety.

### 2.2. Data collection procedure

All participants completed the study instrument electronically via WhatsApp, at their convenience.

### 2.3. Study instrument

The survey package comprised 3 components:

1. **Researcher-developed questionnaire** capturing demographics (e.g., age, sex, education) and clinical characteristics (disease duration, medications, level of physical disability, motor, and non-motor symptoms).
2. **Patient Health Questionnaire-9 (PHQ-9) – Arabic version** to screen for and grade depression severity. It consists of 9 items (total score 0–27); higher scores indicate more severe depressive symptoms. The PHQ-9 demonstrates excellent test–retest reliability and internal consistency (α ≈ 0.86–0.89). Severity cutoffs: 5–9 mild, 10–14 moderate, 15–19 moderately severe, 20–27 severe; a score > 10 yields sensitivity/specificity of 88%/88% for major depression.^[22–24]^ The Arabic PHQ-9 (Alhadi et al.) showed good internal consistency (α = 0.857; inter-item *R* = 0.177–0.648).^[22]^
3. **Generalized Anxiety Disorder-7 (GAD-7) – Arabic version** to screen for and grade generalized anxiety. It includes 7 items scored 0–3 (total 0–21); higher scores indicate greater anxiety. The GAD-7 has excellent internal consistency (α = 0.93) and good test–retest reliability (ICC = 0.83). Severity cutoffs: 0–4 minimal, 5–9 mild, 10–14 moderate, 15–21 severe.^[25]^ The Arabic GAD-7 (Alhadi et al) showed acceptable internal consistency (α = 0.763; inter-item *R* = 0.204–0.426).^[22]^

### 2.4. Permissions

Use of the Arabic PHQ-9, and GAD-7 was authorized; all necessary permissions were obtained from the respective rights holders/authors.

### 2.5. Ethical consideration

The study was approved by the Institutional Review Board (IRB) at the College of Medicine, King Saud University, Riyadh, Saudi Arabia (research project no. E-24-9333). All participants in the study agreed to participate voluntarily, and their information was kept strictly confidential.

### 2.6. Statistical analysis

Statistical analysis was performed in SPSS version 28 (IBM Co., Armonk, NY, USA). Numerical data were presented as the mean and standard deviation (SD). Categorical data were presented as the frequency and percentage and analyzed using the chi-square test or the exact test, as appropriate. Logistic regression analyses were performed to assess factors associated with depression and anxiety. Age and disease duration were analyzed as categorical variables; therefore, formal normality testing was not required for the primary analyses, and mean ± standard deviation values are presented for descriptive purposes only. A two-tailed *P*-value < 0.05 was considered statistically significant.

## 3. Results

### 3.1. Sociodemographic data of the study participants

Table 1 presents the demographic and clinical characteristics of the study population. The study included 130 patients with Parkinson’s disease (90 male and 40 female), with 32.31% of them belonging to the 51–60-year-old group and 30% belonging to the 61–70-year-old group. Most were married (74.62%) and unemployed (68.46%). The highest educational level was a bachelor’s degree (37.69% of patients) and high school or below (31.54%).

**Table 1:** Sociodemographic data of the study participants.

Item	Total participants (N* *= 130)
**Age (years**)	
40–50	21 (16.15%)
51–60	42 (32.31%)
61–70	39 (30.00%)
71–80	16 (12.31%)
>80	12 (9.23%)
**Gender**	
Male	90 (69.23%)
Female	40 (30.77%)
**Marital status**	
Married	97 (74.62%)
Divorced/separated	12 (9.23%)
Widowed	15 (11.54%)
Single	6 (4.62%)
**Highest educational level**	
Master’s degree/PhD	17 (13.08%)
Bachelor’s degree	49 (37.69%)
High school or below	41 (31.54%)
Uneducated	23 (17.69%)
**Employment status**	
Unemployed	89 (68.46%)
Employed	41 (31.54%)

Numerical data are presented as mean ± SD, and categorical data are presented as frequency (%).

SD = standard deviation.

### 3.2. Psychiatric-related data of the study participants

Psychiatric-related data are descriptively depicted in Table 2, which shows that 24.62% of patients were previously diagnosed with major depressive disorder, 20.77% with generalized anxiety disorder, and 9.23% with other psychiatric illnesses. Twenty-five patients reported being on psychotropic medications, of whom 56% were on antidepressants and anti medications, 20% on antipsychotic medications, and 4% on mood stabilizers. More than a third of patients (34.62%) were first diagnosed with PD 3 to 5 years ago, and 27.69% were diagnosed 5 to 10 years ago. One hundred and twenty patients were on PD medications, with levodopa/carbidopa (Sinemet) being the predominant one taken (92.5%), followed by dopamine agonists (e.g., pramipexole, ropinirole) (32.5%). Moreover, 7.69% of patients underwent deep brain stimulation (DBS), and 5.38% had a Duodopa® pump for PD. Over the previous 2 weeks, 72.31% of patients manifested slowness in movement and walking, 70.77% had hand tremors, 43.85% had balance problems or falls while walking, and 42.31% had constant pain in a part of the body.

**Table 2:** Psychiatric-related data of the studied participants.

Item	Total participants (N* *= 130)
**Have you ever been diagnosed with major depressive disorder?**	
No	98 (75.38%)
Yes	32 (24.62%)
**Have you ever been diagnosed with generalized anxiety disorder?**	
No	103 (79.23%)
Yes	27 (20.77%)
**Have you been diagnosed with any other psychiatric illness(es)?**	
No	118 (90.77%)
Yes	12 (9.23%)
**Are you currently taking medications for depression or anxiety?**	
No	105 (80.77%)
Yes	25 (19.23%)
**Psychotropic medications taken** *More than 1 answer is allowed	(n = 25)
Antidepressants and antianxiety medications	14 (56.00%)
Antipsychotic medications	5 (20.00%)
Mood stabilizers	1 (4.00%)
Other	7 (28.00%)
**When were you first diagnosed with Parkinson’s disease?**	
<3 years ago	22 (16.92%)
3–5 years ago	45 (34.62%)
5–10 years ago	36 (27.69%)
10–15 years ago	13 (10.00%)
More than 15 years ago	14 (10.77%)
**Are you currently taking medications for Parkinson’s disease?**	
No	10 (7.69%)
Yes	120 (92.31%)
**Parkinson’s disease medications taken** *More than 1 answer is allowed	(n = 120)
Levodopa/carbidopa (Sinemet)	111 (92.5%)
Dopamine agonists (e.g., pramipexole, ropinirole)	39 (32.5%)
COMT inhibitors (e.g., entacapone)	10 (8.33%)
Amantadine	16 (13.33%)
Anticholinergics (e.g., benztropine)	2 (1.67%)
Other	3 (2.50%)
**Have you undergone any advanced therapies for Parkinson’s disease?**	
No	113 (86.92%)
Yes, deep brain stimulation (DBS)	10 (7.69%)
Yes, Duodopa® pump	7 (5.38%)
**Over the past 2 weeks, have you experienced any of the following symptoms?**	
Tremor in the hands	92 (70.77%)
Slowness in movement and walking	94 (72.31%)
Balance problems or falling while walking	57 (43.85%)
Difficulty in speaking	52 (40.00%)
Drooling	40 (30.77%)
Difficulty in swallowing	23 (17.69%)
Difficulty staying awake during the day	25 (19.23%)
Inability to control urine	45 (34.62%)
Severe or persistent constipation	35 (26.92%)
Constant pain in a part of the body	55 (42.31%)
Dizziness or feeling faint (lightheaded) when standing	32 (24.62%)
Difficulty performing daily tasks (such as eating, dressing)	44 (33.85%)
I do not suffer from any of the abovementioned symptoms	2 (1.54%)

Numerical data are presented as mean ± SD, and categorical data are presented as frequency (%).

COMT = catechol-O-methyltransferase, DBS = deep brain stimulation.

### 3.3. PHQ-9 and GAD-7

**Figure 1.**
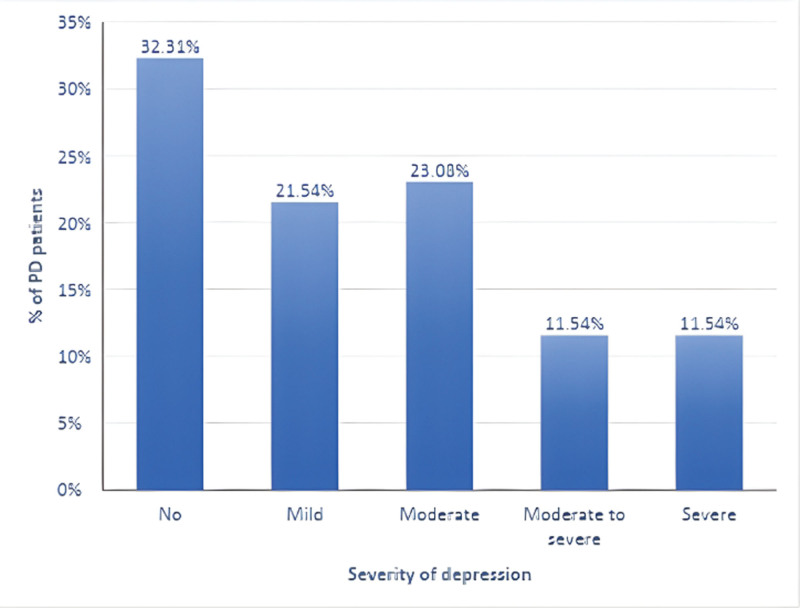
Distribution of patients with Parkinson’s disease according to the severity of depression.

The proportions answering “not at all” (over the previous 2 weeks) on each PHQ-9 item were as follows: little interest/pleasure, 36.15%; feeling down/depressed/hopeless, 36.92%; sleep problems, 26.15%; fatigue, 15.38%; poor appetite/overeating, 41.54%; negative self-perception/feeling like a failure, 62.31%; trouble concentrating, 62.31%; psychomotor change (slowness or restlessness), 34.62%; and thoughts of self-harm, 80.00%. Most patients had at least some sleep disturbance (73.85%) and fatigue (84.62%). These problems made functioning not difficult for 23.85% of participants, somewhat difficult for 46.15%, very difficult for 17.69%, and extremely difficult for 12.31%. Overall PHQ-9 severity was as follows: no depression, 32.31%; mild, 21.54%; moderate, 23.08%; moderate to severe, 11.54%; and severe, 11.54% (mean 9.28 ± 7.1) (Table 3, Figure 1).

**Table 3:** Patient health questionnaire-9 (PHQ-9).

Item	Not at all	Several days	More than half the days	Nearly every day
**Over the last 2 weeks, how often have you been bothered by any of the following problems?**				
Little interest or pleasure in doing things	47 (36.15%)	34 (26.15%)	21 (16.15%)	28 (21.54%)
Feeling down, depressed, or hopeless	48 (36.92%)	44 (33.85%)	18 (13.85%)	20 (15.38%)
Trouble falling or staying asleep, or sleeping too much	34 (26.15%)	47 (36.15%)	19 (14.62%)	30 (23.08%)
Feeling tired or having little energy	20 (15.38%)	43 (33.08%)	25 (19.23%)	42 (32.31%)
Poor appetite or overeating	54 (41.54%)	42 (32.31%)	20 (15.38%)	14 (10.77%)
Feeling bad about yourself, or that you are a failure, or have let yourself or your family down	81 (62.31%)	19 (14.62%)	16 (12.31%)	14 (10.77%)
Trouble concentrating on things, such as reading the newspaper or watching television	81 (62.31%)	22 (16.92%)	12 (9.23%)	15 (11.54%)
Moving or speaking so slowly that other people could have noticed? Or the opposite – being so fidgety or restless that you have been moving around a lot more than usual	45 (34.62%)	38 (29.23%)	17 (13.08%)	30 (23.08%)
Thoughts that you would be better off dead or of hurting yourself in some way	104 (80.00%)	17 (13.08%)	2 (1.54%)	7 (5.38%)
	**Not difficult at all**	**Somewhat difficult**	**Very difficult**	**Extremely difficult**
**How difficult have these problems made it for you to do your work, take care of things at home, or get along with other people?**	31 (23.85%)	60 (46.15%)	23 (17.69%)	16 (12.31%)
Total score	9.28 ± 7.1			
Depression				
No depression	42 (32.31%)			
Mild	28 (21.54%)			
Moderate	30 (23.08%)			
Moderate to severe	15 (11.54%)			
Severe	15 (11.54%)			

Numerical data are presented as mean ± SD, and categorical data are presented as frequency (%).

PHQ-9 = patient health questionnaire-9, SD = standard deviation.

**Figure 2.**
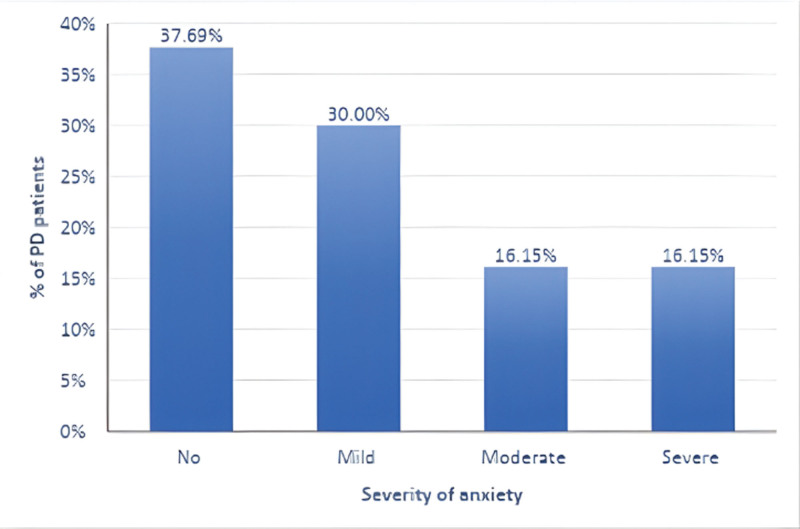
Distribution of patients with Parkinson’s disease according to the severity of anxiety.

GAD-7 item responses showed that for several days over the previous 2 weeks 39.23% of the participants felt “nervous/anxious/on edge” and 35.38% were “worrying too much about different things.” The proportions answering “not at all” were as follows: cannot control worrying, 43.85%; trouble relaxing, 35.38%; restlessness, 44.62%; irritability, 44.62%; and feeling afraid that something awful might happen, 50.77%. These problems made functioning somewhat difficult for 43.85% of patients. Overall, GAD-7 severity was as follows: no anxiety, 37.69%; mild, 30.00%; moderate, 16.15%; severe, 16.15% (mean 7.37 ± 6.2) (Table 4, Figure 2).

**Table 4:** Generalized anxiety disorder-7 (GAD-7).

Item	Not at all	Several days	More than half the days	Nearly every day
Over the last 2 weeks, how often have you been bothered by any of the following problems?				
Feeling nervous, anxious, or on edge	42 (32.31%)	51 (39.23%)	19 (14.62%)	18 (13.85%)
Not being able to stop or control worrying	57 (43.85%)	40 (30.77%)	16 (12.31%)	17 (13.08%)
Worrying too much about different things	43 (33.08%)	46 (35.38%)	16 (12.31%)	25 (19.23%)
Trouble relaxing	46 (35.38%)	34 (26.15%)	21 (16.15%)	29 (22.31%)
Being so restless that it is hard to sit still	58 (44.62%)	33 (25.38%)	16 (12.31%)	23 (17.69%)
Becoming easily annoyed or irritable	58 (44.62%)	35 (26.92%)	22 (16.92%)	15 (11.54%)
Feeling afraid, as if something awful might happen	66 (50.77%)	27 (20.77%)	20 (15.38%)	17 (13.08%)
	**Not difficult at all**	**Somewhat difficult**	**Very difficult**	**Extremely difficult**
How difficult have they made it for you to do your work, take care of things at home, or get along with other people?	33 (25.38%)	57 (43.85%)	20 (15.38%)	20 (15.38%)
Total score	7.37 ± 6.2			
Anxiety				
No anxiety	49 (37.69%)			
Mild	39 (30.00%)			
Moderate	21 (16.15%)			
Severe	21 (16.15%)			

Numerical data are presented as mean ± SD, and categorical data are presented as frequency (%).

GAD-7 = Generalized Anxiety Disorder-7, SD = standard deviation.

### 3.4. Regression analysis

A significant relation was found between the depression and anxiety of patients and both age and educational level: 35.71% of non patients versus 30.68% of depressed patients were 51 to 60 years old, and 42.86% versus 23.86% were 61 to 70 years old (*P *= .013); 28.57% versus 42.05% had bachelor’s degrees, and 28.57% versus 32.95% had high school education or below (*P *= .021). Moreover, 38.78% of non-anxious patients versus 28.4% of anxious patients were 51 to 60 years old, and 36.73% versus 25.93% were 61 to 70 years old (*P *= .008); 30.61% versus 41.98% had bachelor’s degrees, and 32.65% versus 30.86% had a high school education or below (*P *= .017) (Table 5).

**Table 5:** Relation between sociodemographic data of patients with Parkinson’s disease and those with depression and anxiety.

Item	With depression		*p*-value	With anxiety		*p*-value
	No (n* *= 42)	Yes (n* *= 88)		No (n* *= 49)	Yes (n* *= 81)	
Age (years)						
40–50	1 (2.38%)	20 (22.73%)	**.013**	2 (4.08%)	19 (23.46%)	**.008**
51–60	15 (35.71%)	27 (30.68%)		19 (38.78%)	23 (28.4%)	
61–70	18 (42.86%)	21 (23.86%)		18 (36.73%)	21 (25.93%)	
71–80	3 (7.14%)	13 (14.77%)		3 (6.12%)	13 (16.05%)	
>80	5 (11.90%)	7 (7.95%)		7 (14.29%)	5 (6.17%)	
Gender						
Male	32 (76.19%)	58 (65.91%)	.235	35 (71.43%)	55 (67.90%)	.673
Female	10 (23.81%)	30 (34.09%)		14 (28.57%)	26 (32.10%)	
Marital status						
Married	33 (78.57%)	64 (72.73%)	.119	38 (77.55%)	59 (72.84%)	.209
Divorced/separated	6 (14.29%)	6 (6.82%)		6 (12.24%)	6 (7.41%)	
Widowed	3 (7.14%)	12 (13.64%)		5 (10.20%)	10 (12.35%)	
Single	0 (0.00%)	6 (6.82%)		0 (0.00%)	6 (7.41%)	
Highest educational level						
Master’s degree/PhD	11 (26.19%)	6 (6.82%)	**.021**	12 (24.49%)	5 (6.17%)	**.017**
Bachelor’s degree	12 (28.57%)	37 (42.05%)		15 (30.61%)	34 (41.98%)	
High school or below	12 (28.57%)	29 (32.95%)		16 (32.65%)	25 (30.86%)	
Uneducated	7 (16.67%)	16 (18.18%)		6 (12.24%)	17 (20.99%)	
Employment status						
Unemployed	28 (66.67%)	61 (69.32%)	.761	32 (65.31%)	57 (70.37%)	.547
Employed	14 (33.33%)	27 (30.68%)		17 (34.69%)	24 (29.63%)	

Numerical data are presented as mean ± SD, and categorical data are presented as frequency (%). Statistical significance at *P*-value < .05.

SD = standard deviation.

As shown in Table 6, having depression according to PHQ-9 was significantly associated with a positive history of major depressive disorder (11.9% of nondepressed patients vs 30.68% of depressed patients had positive history, *P *= .020), time of diagnosis with PD (42.86% vs 30.68% were diagnosed 3–5 years ago, and 21.43% vs 30.68% were diagnosed 5–10 years ago, *P *< .001), and not taking medications for it (0% vs 11.36% were not taking medications, *P *= .023). Furthermore, having anxiety according to GAD-7 was significantly associated with a positive history of major depressive disorder (10.20% of non-anxious patients vs 33.33% of anxious patients had positive history, *P *= .003) and time of diagnosis with PD (40.82% vs 30.86% were diagnosed 3–5 years ago, and 20.41% vs 32.1% were diagnosed 5–10 years ago, *P *= .001).

**Table 6:** Relation between psychiatric-related data of patients with Parkinson’s disease and those with depression and anxiety.

Item	With depression		*p*-value	With anxiety		*p*-value
	No (n* *= 42)	Yes (n* *= 88)		No (n = 49)	Yes (n = 81)	
Have you ever been diagnosed with major depressive disorder?						
No	37 (88.10%)	61 (69.32%)	**.020**	44 (89.80%)	54 (66.67%)	**.003**
Yes	5 (11.90%)	27 (30.68%)		5 (10.20%)	27 (33.33%)	
Have you ever been diagnosed with generalized anxiety disorder?						
No	35 (83.33%)	68 (77.27%)	.426	43 (87.76%)	60 (74.07%)	.062
Yes	7 (16.67%)	20 (22.73%)		6 (12.24%)	21 (25.93%)	
Have you been diagnosed with any other psychiatric illness(es)?						
No	41 (97.62%)	77 (87.50%)	.062	47 (95.92%)	71 (87.65%)	.115
Yes	1 (2.38%)	11 (12.50%)		2 (4.08%)	10 (12.35%)	
Are you currently taking medications for depression or anxiety?						
No	38 (90.48%)	67 (76.14%)	.052	43 (87.76%)	62 (76.54%)	.116
Yes	4 (9.52%)	21 (23.86%)		6 (12.24%)	19 (23.46%)	
When were you first diagnosed with Parkinson’s disease?						
<3 years ago	14 (33.33%)	8 (9.09%)	**<.001**	15 (30.61%)	7 (8.64%)	**.001**
3–5 years ago	18 (42.86%)	27 (30.68%)		20 (40.82%)	25 (30.86%)	
5–10 years ago	9 (21.43%)	27 (30.68%)		10 (20.41%)	26 (32.1%)	
10–15 years ago	0 (0.00%)	13 (14.77%)		3 (6.12%)	10 (12.35%)	
More than 15 years ago	1 (2.38%)	13 (14.77%)		1 (2.04%)	13 (16.05%)	
Are you currently taking medications for Parkinson’s disease?						
No	0 (0.00%)	10 (11.36%)	**.023**	1 (2.04%)	9 (11.11%)	.060
Yes	42 (100.00%)	78 (88.64%)		48 (97.96%)	72 (88.89%)	
Have you undergone any advanced therapies for Parkinson’s disease?						
No	40 (95.24%)	73 (82.95%)	.068	45 (91.84%)	68 (83.95%)	.439
Yes, deep brain stimulation (DBS)	0 (0.00%)	10 (11.36%)		2 (4.08%)	8 (9.88%)	
Yes, Duodopa® pump	2 (4.76%)	5 (5.68%)		2 (4.08%)	5 (6.17%)	

Numerical data are presented as mean ± SD, and categorical data are presented as frequency (%). Statistical significance at *P*-value < .05.

DBS = deep brain stimulation, SD = standard deviation.

The univariate logistic regression model showed that age, educational level, and history of major depressive disorder were significantly associated with depression of patients with PD: the 51–60, 61–70, and > 80 year-old groups had significantly lower odds of having depression than the 40–50 year-old group, with ORs (95%CI) of 0.09 (0.01–0.74, *P *= .025), 0.06 (0.01–0.48, *P *= .008), and 0.07 (0.01–0.71, *P *= .024), respectively. Compared to patients with a master’s degree/PhD, those with a bachelor’s degree or high school education or below and uneducated patients showed significantly higher odds of depression, with ORs (95%CI) of 5.65 (1.72–18.56, *P *= .004), 4.43 (1.33–14.72, *P *= .015), and 4.19 (1.1–15.9, *P *= .035), respectively. Patients with a history of major depressive disorder showed higher odds of depression according to PHQ-9 than those with no such history (OR = 3.28, 95%CI: 1.16–9.25, *P *= .025).

After adjustment for the included factors, age and educational level were found to have a statistically significant association with depression: the 51–60, 61–70, and > 80 year-old groups had significantly lower odds of having depression than the 40–50 year-old group, with ORs (95%CI) of 0.06 (0.01–0.59, *P *= .015), 0.03 (0–0.42, *P *= .008), and 0.04 (0–0.55, *P *= .017), respectively. Compared to patients with a master’s degree/PhD, those with a bachelor’s degree had significantly higher odds of depression (OR = 5.03, 95%CI: 1.2–21.08, *P *= .027) (Table 7).

**Table 7:** Logistic regression analysis for factors associated with depression in patients with Parkinson’s disease.

Item	Univariate analysis			Multivariable analysis		
	Unadjusted OR	95%CI	*p*-value	Adjusted OR	95%CI	*p*-value
Age (years)						
40–50	Ref			Ref		
51–60	0.09	0.01–0.74	**.025**	0.06	0.01–0.59	**.015**
61–70	0.06	0.01–0.48	**.008**	0.03	0–0.42	**.008**
71–80	0.22	0.02–2.31	.206	0.1	0.01–1.57	.101
>80	0.07	0.01–0.71	**.024**	0.04	0–0.55	**.017**
Gender						
Male	Ref			Ref		
Female	1.66	0.72–3.82	.237	2.19	0.71–6.8	.175
Highest educational level						
Master’s degree/PhD	Ref			Ref		
Bachelor’s degree	5.65	1.72–18.56	**.004**	5.03	1.2–21.08	**.027**
High school or below	4.43	1.33–14.72	**.015**	2.97	0.65–13.6	.161
Uneducated	4.19	1.1–15.9	**.035**	1.72	0.29–10.32	.552
Employment status						
Unemployed	Ref			Ref		
Employed	0.89	0.4–1.94	.761	0.38	0.1–1.4	.145
History of major depressive disorder						
No	Ref			Ref		
Yes	3.28	1.16–9.25	**.025**	4.4	0.83–23.19	.081
History of generalized anxiety disorder						
No	Ref			Ref		
Yes	1.47	0.57–3.81	.427	0.75	0.16–3.44	.706
History of any other psychiatric illness(es)						
No	Ref			Ref		
Yes	5.86	0.73–46.97	.096	12.24	0.59–255.84	.106
Taking medications for depression or anxiety						
No	Ref			Ref		
Yes	2.98	0.95–9.32	.061	0.67	0.09–4.99	.696

Statistical significance at *P*-value < .05.

CI = Confidence interval, OR = odds ratio.

The univariate logistic regression model showed that age, educational level, history of major depressive disorder, and time of diagnosis with Parkinson’s disease were significantly associated with anxiety: the 40–50 year-old group had significantly higher odds of having anxiety than the 61–70-year-old group, with an OR (95%CI) of 8.14 (1.67–39.81, *P *= .010). Patients with a bachelor’s degree or high school education or below and uneducated patients had significantly higher odds of anxiety than those with a master’s degree/PhD, with ORs (95%CI) of 5.44 (1.63–18.19, *P *= .006), 3.75 (1.11–12.67, *P *= .033), and 6.8 (1.68–27.52, *P *= .007), respectively. Patients with a history of major depressive disorder had higher odds of anxiety according to GAD-7 than those with no such history (OR = 4.4, 95%CI: 1.56–12.37, *P *= .005). Compared to patients diagnosed with PD <3 years ago, those diagnosed 5–10 and > 10 years ago had higher odds of anxiety, with ORs (95%CI) of 5.57 (1.75–17.7, *P *= .004) and 12.32 (3.07–49.47, *P* < .001), respectively.

In the multivariable model, the 40–50 year-old group had significantly higher odds of having anxiety than the 61–70-year-old group, with an OR (95%CI) of 21.9 (1.73–276.56, *P *= .017). Patients with a history of other psychiatric illness(es) showed significantly higher odds of anxiety than those with no such history (OR = 48.41, 95%CI: 2.51–932.5, *P *= .010). Compared to patients diagnosed with PD <3 years ago, those diagnosed 3–5, 5–10, and > 10 years ago had higher odds of anxiety, with ORs (95%CI) of 6.06 (1.22–30.2, *P *= .028), 14.43 (2.58–80.6, *P *= .003), and 51.14 (4.64–563.61, *P *= .002), respectively (Table 8).

**Table 8:** Logistic regression analysis for factors associated with anxiety in patients with Parkinson’s disease.

Item	Univariate analysis			Multivariable analysis		
	Unadjusted OR	95%CI	*p*-value	Adjusted OR	95%CI	*p*-value
Age (years)						
40–50	8.14	1.67–39.81	**.010**	21.9	1.73–276.56	**.017**
51–60	1.04	0.43–2.49	.934	1.08	0.27–4.37	.909
61–70	Ref			Ref		
71–80	3.71	0.91–15.13	.067	3.54	0.42–30.19	.247
>80	0.61	0.17–2.27	.463	0.22	0.03–1.51	.122
Gender						
Male	Ref			Ref		
Female	1.18	0.54–2.57	.673	0.99	0.3–3.26	.988
Highest educational level						
Master’s degree/PhD	Ref			Ref		
Bachelor’s degree	5.44	1.63–18.19	**.006**	4.48	0.86–23.22	.074
High school or below	3.75	1.11–12.67	**.033**	1.72	0.3–9.94	.543
Uneducated	6.8	1.68–27.52	**.007**	8.67	0.99–76.13	.051
Employment status						
Unemployed	Ref			Ref		
Employed	0.79	0.37–1.69	.547	0.78	0.17–3.53	.746
History of major depressive disorder						
No	Ref			Ref		
Yes	4.4	1.56–12.37	**.005**	5.01	0.7–35.78	.108
History of generalized anxiety disorder						
No	Ref			Ref		
Yes	2.51	0.93–6.74	.068	1.73	0.27–10.98	.562
History of any other psychiatric illness(es)						
No	Ref			Ref		
Yes	3.31	0.69–15.79	.133	48.41	2.51–932.5	**.010**
Taking medications for depression or anxiety						
No	Ref			Ref		
Yes	2.2	0.81–5.95	.122	0.1	0.01–1.0	.050
Time of diagnosis with Parkinson’s disease						
<3 years ago	Ref			Ref		
3–5 years ago	2.68	0.92–7.83	.072	6.06	1.22–30.2	**.028**
5–10 years ago	5.57	1.75–17.7	**.004**	14.43	2.58–80.6	**.002**
More than 10 years ago	12.32	3.07–49.47	**<.001**	51.14	4.64–563.61	**.001**
Taking medications for Parkinson’s disease						
No	Ref			Ref		
Yes	0.17	0.02–1.36	.094	0.85	0.07–10.43	.897
Having any advanced therapies for Parkinson’s disease						
No	Ref			Ref		
Yes, deep brain stimulation (DBS)	2.65	0.54–13.04	.232	0.41	0.04–4.41	.46
Yes, Duodopa® pump	1.65	0.31–8.9	.558	0.75	0.08–7.1	.80

Statistical significance at *P*-value < .05.

CI = confidence interval, DBS = deep brain stimulation, OR = odds ratio.

## 4. Discussion

This study aimed to explore the prevalence and correlates of depression and anxiety among patients with PD in Saudi Arabia. We found a high prevalence of both depression (67.7%) and anxiety (62.3%) among patients with PD. These rates are significantly higher than global estimates from a systematic review, which placed the prevalence of depression in those with PD at 30–40% and anxiety at 20–50%.^[11]^ However, our findings are consistent with an Algerian study that showed similar high rates of approximately 2 thirds for both depression (66.18%) and anxiety (69.70%).^[15]^ Such high rates may partially be explained by the fact that several Parkinsonian features overlap with symptoms captured by anxiety and depression scales (e.g., hypomimia/flat affect, bradykinesia/psychomotor slowing, tremor, muscle tension, fatigue, and autonomic symptoms); screening tools may thus conflate symptom scores and prevalence estimates unless backed by diagnostic interviews.^[6,26]^ This finding highlights the importance of proactive screening and the integration of mental health care in PD management.

Moreover, we found an inverse relation between age and psychiatric symptoms. Younger patients (aged 40–50) had significantly higher odds of depression than older age groups and higher odds of anxiety among than the 61–70 age group. Similar findings were reported in a cross-sectional case series of 79 PD patients: Individuals younger than 62 years were significantly more likely to have anxiety than their older counterparts.^[13]^ Likewise, a cross-sectional study of 64 PD patients found that younger patients had significantly higher odds of having an anxiety disorder than older patients. This difference could be due to the greater psychological burden younger individuals with PD face as a result of disrupted careers, loss of independence, and increased family responsibilities.^[8]^ This finding reinforces the importance of early detection and screening of depression and anxiety in PD patients.

Furthermore, our study showed a statistically significant association between lower educational attainment and higher rates of depression and anxiety. Direct comparisons of educational level and rates of depression and anxiety in PD patients are limited in the literature; to the best of our knowledge, no published studies have examined the association. One possible explanation for our findings is that lower educational attainment may be associated with reduced health literacy or engagement with treatment plans. This explanation aligns with a cross-sectional study of 168 PD patients, which found that those with a high school education or less had significantly lower health literacy, potentially limiting their engagement with treatment plans.^[27]^ In addition, a cross-sectional national survey of 769 PD patients found that lower education was significantly associated with greater perceived barriers to understanding and accessing mental health care.^[28]^ Clinically, this finding highlights the need to adapt mental health support based on patients’ education level, by using clear communication, simplifying treatment instructions, and providing extra guidance for vulnerable groups.

Additionally, our study showed a strong association between longer PD duration and anxiety: compared with patients diagnosed < 3 years ago, those diagnosed 3–5, 5–10, and > 10 years ago had higher odds of anxiety. This finding is consistent with a prospective, hospital-based observational study of 105 PD patients, which reported that more than half of the participants experienced anxiety, and its prevalence increased significantly with longer disease duration.^[29]^ Similarly, in another cross-sectional observational study of 66 PD patients, it was found that longer disease duration was significantly associated with higher anxiety scores.^[30]^ Future longitudinal studies are needed to explore how the trajectory of PD influences the onset and progression of anxiety over time.

Furthermore, we identified a previous history of psychiatric illness as a key contributor to current depressive and anxiety symptoms. This aligns with a cross-sectional study of 79 PD patients, which reported that those with a previous psychiatric history were significantly more likely to experience anxiety disorders, with the odds increased nearly fivefold.^[13]^ Clinically, this finding underscores the importance of considering previous psychiatric history when developing individualized care plans for PD patients.

Interestingly, patients who were not on medications for PD were more likely to report depressive symptoms; however, the number of untreated patients in our sample was small (n = 10), which may have influenced this finding. Direct comparisons of untreated PD status and depression are limited in the literature; no published studies have specifically examined the association between being off PD pharmacotherapy and depressive symptoms. However, related research indicates that depression in PD is linked to treatment non‑adherence: In a cross-sectional study including 51 PD patients, those with longer disease duration and depression also showed significantly lower adherence to PD medications.^[31]^ This suggests the need for further research using larger, more representative samples to better understand the relation between pharmacological treatment and psychiatric outcomes in PD.

Lastly, we did not find significant associations between gender, marital status, or employment status and the presence of depression or anxiety. This is in contrast to a cross-sectional study of 64 PD patients, which found that depression was significantly more prevalent among female patients and those of low socioeconomic status.^[8]^ Similarly, a pooled cross-sectional study of 1509 PD patients, of whom 571 were women, found that women had significantly higher anxiety scores than men, indicating greater prevalence or severity of anxiety among female patients.^[32]^ As for marital status, direct comparisons with rates of depression and anxiety in PD patients are limited in the literature. Our inability to find a significant association between gender, marital status, or employment status and the presence of depression or anxiety may be due to the small sample, which may have limited the statistical power to detect such associations. Future research involving larger, more diverse samples may provide deeper insight into how demographic factors influence psychiatric outcomes in PD.

## 5. Study strengths, limitations, and future directions

To our knowledge, this is the largest and one of the few studies addressing psychiatric comorbidities in PD patients in Saudi Arabia and thus offers novel insights into the care of PD patients in the region. Beyond prevalence, our study examined underlying contributory factors in an effort to identify targetable interventions in the care of PD patients. Identifying at-risk groups highlights those who would benefit from regular screening to prevent depression and anxiety. This study also has several limitations. First, it was primarily designed to measure the prevalence of depression and anxiety in PD, so it may lack the power to detect other important predictors (risk of type II error). Second, recruiting patients from a single tertiary center limits external validity and introduces Berkson’s bias, potentially inflating the observed prevalence of mood disorders. The latter limitation, however, was partially mitigated through additional recruitment from the Saudi Parkinson’s Society. Including an appropriate control group in future studies would reduce this bias and allow a more accurate estimate of the burden attributable to Parkinson’s disease.

## 6. Conclusion

Our study found a high prevalence of mood symptoms in PD (depression 67.7%, anxiety 62.3%). Except for one Algerian series, these rates exceed those reported in other international cohorts and suggest a heavier local burden.^[15]^ Additionally, prolonged disease duration, younger age, and lower educational levels were significantly associated with having depression and/or anxiety. Routine neuropsychiatric screening and an early, multidisciplinary approach should be integrated into PD care.

## Acknowledgments

The authors thank Dr Ahmad N. AlHadi and colleagues for developing and generously providing the validated Arabic version of the Patient Health Questionnaire (PHQ-9) used in this study, and for granting permission to use the instrument.

## Author contributions

**Conceptualization:** Sohaila Alshimemeri, Mohammed A. Aljaffer, Ayedh H. Alghamdi, Naif Alsaber, Ahmad H. Almadani.

**Data curation:** Sohaila Alshimemeri, Ayedh H. Alghamdi, Ahmad H. Almadani.

**Formal analysis:** Sohaila Alshimemeri, Ayedh H. Alghamdi, Ahmad H. Almadani.

**Writing – review & editing:** Sohaila Alshimemeri, Mohammed A. Aljaffer, Ayedh H. Alghamdi, Ahmad H. Almadani.

**Writing – original draft:** Nouf F. Alshammari, Naif Alsaber, Rakan S. Alromayan, Sohaila Alshimemeri, Ahmad H. Almadani.

**Investigation:** Rakan S. Alromayan.

## References

[1] Foltynie T Brayne C Barker RA. The heterogeneity of idiopathic Parkinson’s disease. J Neurol. 2002;249:138–45. 11985378 10.1007/pl00007856

[2] Parkinson Disease: A Public Health Approach. Technical Brief. 1st ed. World Health Organization; 2022.

[3] Maserejian NN Vinikoor-Imler LC Dilley A. Estimation of the 2020 global population of Parkinson’s disease (PD) [Abstract]. Mov Disord. 2020;35(Suppl 1).

[4] Safiri S Noori M Nejadghaderi SA. The burden of Parkinson’s disease in the Middle East and North Africa region, 1990-2019: results from the global burden of disease study 2019. BMC Public Health. 2023;23:107. 36642724 10.1186/s12889-023-15018-x PMC9841703

[5] Alamri Y MacAskill M Anderson T Benamer H. Parkinson’s disease in the gulf countries: an updated review. Eur Neurol. 2015;74:222–5. 26613525 10.1159/000442283

[6] Schrag A Barone P Brown RG. Depression rating scales in Parkinson’s disease: critique and recommendations. Mov Disord. 2007;22:1077–92. 17394234 10.1002/mds.21333 PMC2040268

[7] Xiong P Liu M Liu B Hall BJ. Trends in the incidence and DALYs of anxiety disorders at the global, regional, and national levels: estimates from the global burden of disease study 2019. J Affect Disord. 2022;297:83–93. 34678404 10.1016/j.jad.2021.10.022

[8] Reddy MS. Depression: the disorder and the burden. Indian J Psychol Med. 2010;32:1–2. 21799550 10.4103/0253-7176.70510 PMC3137804

[9] Crown S. Psychosomatic aspects of Parkinsonism. J Psychosom Res. 1971;15:451–9. 4941482 10.1016/0022-3999(71)90027-4

[10] Khedr EM Abdelrahman AA Elserogy Y Zaki AF Gamea A. Depression and anxiety among patients with Parkinson’s disease: frequency, risk factors, and impact on quality of life. Egypt J Neurol Psychiatry Neurosurg. 2020;56:116.

[11] Reijnders JSAM Ehrt U Weber WEJ Aarsland D Leentjens AFG. A systematic review of prevalence studies of depression in Parkinson’s disease. Mov Disord. 2008;23:183–9; quiz 313. 17987654 10.1002/mds.21803

[12] Cong S Xiang C Zhang S Zhang T Wang H Cong S. Prevalence and clinical aspects of depression in Parkinson’s disease: a systematic review and meta‑analysis of 129 studies. Neurosci Biobehav Rev. 2022;141:104749. 35750224 10.1016/j.neubiorev.2022.104749

[13] Dissanayaka NNW Sellbach A Matheson S. Anxiety disorders in Parkinson’s disease: prevalence and risk factors. Mov Disord. 2010;25:838–45. 20461800 10.1002/mds.22833

[14] Ghaddar A Fawaz M Khazen G Abdallah J Milane A. Prevalence of depression in Parkinson’s disease in a Lebanese tertiary clinic. J Clin Exp Neuropsychol. 2016;38:51–8. 26588047 10.1080/13803395.2015.1087466

[15] Belarbi S Ouali M Mokrane SM. Clinical and neuropsychological profile in idiopathic Parkinson’s disease: about an Algerian hospital series. IOSR J Dent Med Sci. 2022;21:26–33.

[16] Kolsi S Fakhfakh E Messedi N. Anxiety and depression in tunisian patients with Parkinson’s disease. Eur. Psychiatr. 2023;66:S961–S961.

[17] Tibar H El Bayad K Bouhouche A. Non-motor symptoms of Parkinson’s disease and their impact on quality of life in a cohort of moroccan patients. Front Neurol. 2018;9:170. 29670566 10.3389/fneur.2018.00170 PMC5893866

[18] Shakeri J Chaghazardi M Abdoli N Arman F Hoseini SD Shakeri H. Disease-related variables and depression among Iranian patients with Parkinson disease. Iran Red Crescent Med J. 2015;17:e30246. 26568863 10.5812/ircmj.30246 PMC4636748

[19] Al-Qahtani Z Alqahtani ASM Alzuhairi AM. The prevalence of depression among Parkinson’s disease patients in Saudi Arabia: a cross-sectional study. NDT. 2025; Volume 21:241–56. 10.2147/NDT.S493629 PMC11806747 39926117

[20] Koenig HG Al Zaben F Sehlo MG. Mental health care in Saudi Arabia: past, present and future. OJPsych. 2014;04:113–30.

[21] Al-Krenawi A Graham JR. Culturally sensitive social work practice with Arab clients in mental health settings. Health Soc Work. 2000;25:9–22. 10689599 10.1093/hsw/25.1.9

[22] AlHadi AN AlAteeq DA Al-Sharif E. An arabic translation, reliability, and validation of patient health questionnaire in a Saudi sample. Ann Gen Psychiatry. 2017;16:32. 28878812 10.1186/s12991-017-0155-1 PMC5585978

[23] Kroenke K Spitzer RL Williams JBW. The PHQ-9: validity of a brief depression severity measure. J Gen Intern Med. 2001;16:606–13. 11556941 10.1046/j.1525-1497.2001.016009606.x PMC1495268

[24] Spitzer RL Williams JBW Kroenke K Hornyak R McMurray J. Validity and utility of the PRIME-MD patient health questionnaire in assessment of 3000 obstetric-gynecologic patients: the PRIME-MD patient health questionnaire obstetrics-gynecology study. Am J Obstet Gynecol. 2000;183:759–69. 10992206 10.1067/mob.2000.106580

[25] Spitzer RL Kroenke K Williams JBW Löwe B. A brief measure for assessing generalized anxiety disorder: the GAD-7. Arch Intern Med. 2006;166:1092–7. 16717171 10.1001/archinte.166.10.1092

[26] Leentjens AFG Dujardin K Marsh L. Anxiety rating scales in Parkinson’s disease: critique and recommendations. Mov Disord. 2008;23:2015–25. 18792121 10.1002/mds.22233

[27] Fleisher JE Shah K Fitts W Dahodwala NA. Associations and implications of low health literacy in Parkinson’s disease. Mov Disord Clin Pract. 2016;3:250–6. 27331078 10.1002/mdc3.12272 PMC4909149

[28] Dobkin RD Rubino JT Friedman J Allen LA Gara MA Menza M. Barriers to mental health care utilization in Parkinson’s disease. J Geriatr Psychiatry Neurol. 2013;26:105–16. 23589410 10.1177/0891988713481269 PMC3644337

[29] Upneja A Paul BS Jain D Choudhary R Paul G. Anxiety in Parkinson’s disease: correlation with depression and quality of life. J Neurosci Rural Pract. 2021;12:323–8. 33986584 10.1055/s-0041-1722840 PMC8110433

[30] Foster PS Drago V Crucian GP. Anxiety and depression severity are related to right but not left onset Parkinson’s disease duration. J Neurol Sci. 2011;305:131–5. 21420691 10.1016/j.jns.2011.02.023

[31] Samanta S Bisht M Kanimozhi M Kumar N Handu SS. Association of depression with disease duration, quality of life and adherence in Parkinson’s disease: a cross sectional study. J Family Med Prim Care. 2023;12:1406–11. 37649742 10.4103/jfmpc.jfmpc_2288_22 PMC10465023

[32] Maas BR Göttgens I Tijsse Klasen HPS. Age and gender differences in non-motor symptoms in people with Parkinson’s disease. Front Neurol. 2024;15:1339716. 38361642 10.3389/fneur.2024.1339716 PMC10867965

